# Supplementary material for: Early Permissiveness of Central Nervous System Cells to Measles Virus Infection Is Determined by Hyperfusogenicity and Interferon Pressure
Source: Viruses. 2023 Jan 13;15(1):229. doi: 10.3390/v15010229 (PMC9861295; doi:10.3390/v15010229)
Supplement: Supplementary file 1 [file viruses-15-00229-s001.zip › viruses-1873195-supplementary.pdf]

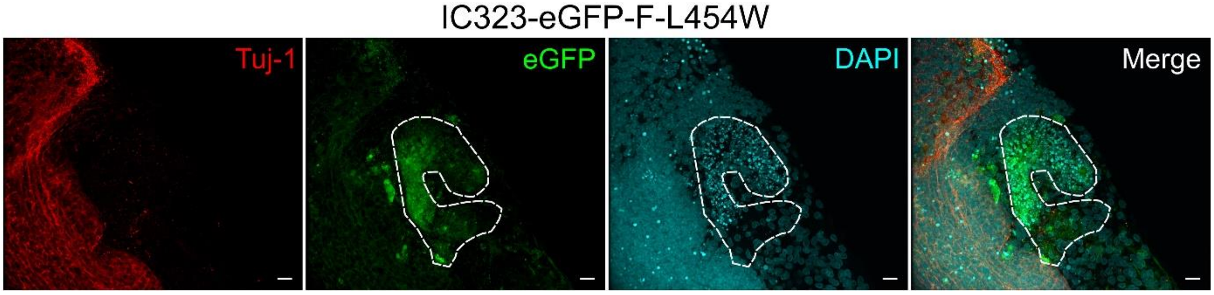

Supplementary Figure S1-Typical example of syncytium observed in hamster cerebellar organotypic cultures. OCC was infected with 2.5×10^4 PFU the day of slice preparation. OCC was stained for neuron (Tuj-1) nuclei (DAPI and infection is observed by eGFP fluorescence. Pictures were obtained by spinning confocal microscopy (CQ1 Yokogawa). Scale Bar = 20μm.

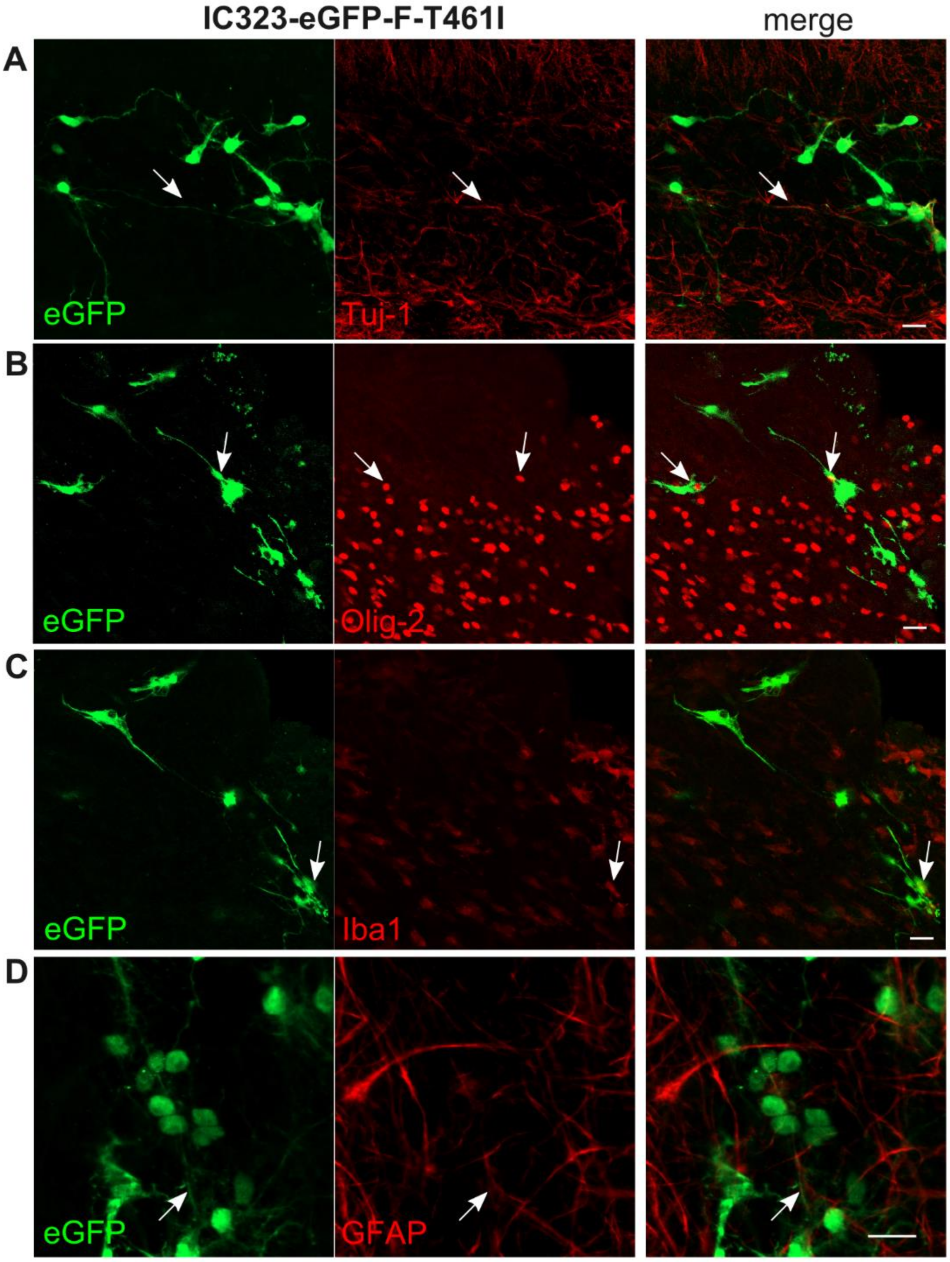

Supplementary Figure S2. Early tropism of MeV IC323-eGFP-F-T461I infection in hamster OCC. The OCCs were infected with 10^4 PFU of the MeV IC323-eGFP-F-T461I virus on the day of the section and fixed with 4% formaldehyde 24 hours post infection. (**A**) Immunofluorescent labeling of tubulin present in neurons (Beta-III-Tubulin, clone Tuj-1), (**B**) of oligodendrocytes engaged cells (Olig-2), (**C**) of microglial cells (iba1) and (**D**) of astrocytes (GFAP) Green fluorescence corresponds to infected cells with virus expressing eGFP. The images were taken with the Zeiss Axio Observer microscope with the LSM 980 confocal system and analyzed with the ImageJ software. The arrows point to the areas of co-localization between the infected cells and the labeling of interest. Scale bar = 20µm.

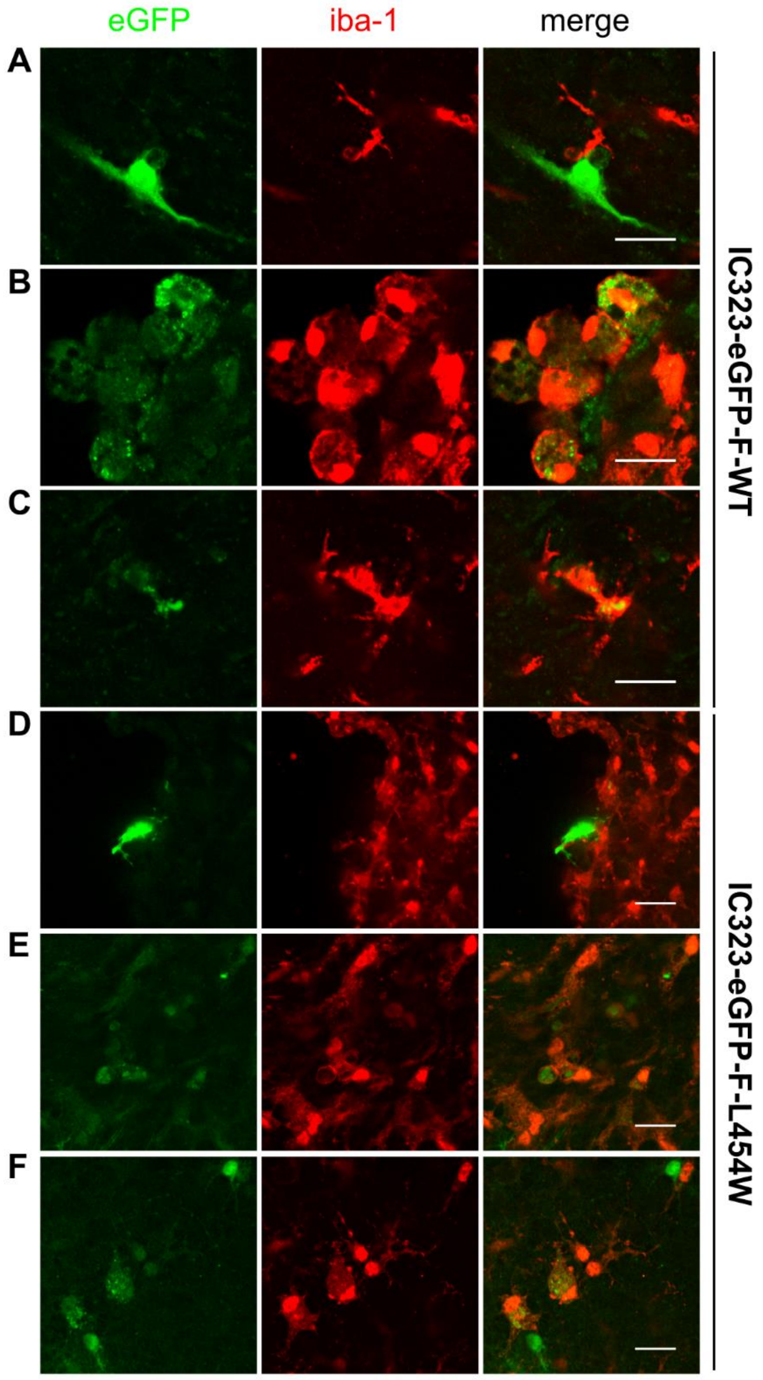

<u>Supplementary Figure S3.</u> **Microglial cells appear to carry out phagocytosis of infected cells in OCCs.** OCCs were infected with (**A-C**) MeV IC323-EGFP-F-WT or (**D-F**) MeV IC323-EGFP- F-L454W after 7 days of culture and analyzed as described in Figures 6 . (**A,D**) Immunofluorescent (IF) staining possibly showing early stage of phagocytosis by iba-1-positive microglial cells surrounding and starting to engulf infected eGFP-positive cells. (**B,C,E,F**) IF staining of microglial cells containing dot-shaped eGFP signal potentially consisting of debris of infected cells that were phagocytosed. Scale bar = 20 μm.

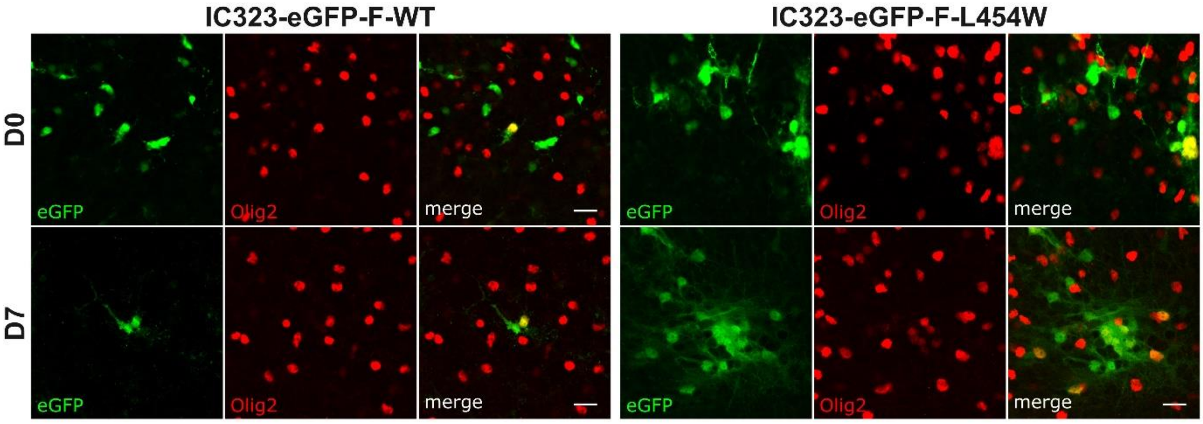

Supplementary Figure S4: Olig2-positive cells are susceptible to MeV infection in OCCs, both at Days 0 and 7 of culture. Oligodendrocyte engaged cells (precursors and differentiated cells) were stained for Olig2 marker. eGFP shows infected cells. Note that Olig2 staining can be found in syncytia observed in MeV IC323-eGFP-F-L454W- infected OCCs both when infected at day 0 or 7 of culture. Pictures were obtained by spinning confocal microscopy (CQ1 Yokogawa). Scale Bar = 20µm.
